# Socio-economic differences in accessing NHS spectacles amongst children with differing refractive errors living in Scotland

**DOI:** 10.1038/s41433-021-01536-8

**Published:** 2021-04-19

**Authors:** Stephanie Kearney, Niall C. Strang, Jim Lewsey, Augusto Azuara-Blanco, Sven Jonuscheit

**Affiliations:** 1 Department of Vision Sciences, Glasgow Caledonian University, Glasgow, UK; 2 Institute of Health and Wellbeing, University of Glasgow, Glasgow, UK; 3 Centre for Public Health, Queen’s University Belfast, Belfast, UK

**Keywords:** Epidemiology, Epidemiology

## Abstract

**Background/objectives:**

Adults living in more deprived areas are less likely to attend an eye examination, resulting in greater visual impairment from undiagnosed eye disease and a widening of health inequalities. It is unknown if the introduction of free NHS eye examinations and help with spectacle costs has benefited children in Scotland. This study aimed to explore factors associated with accessing NHS spectacles including level of deprivation, refractive error, urbanity and age.

**Subjects/methods:**

NHS-financed General Ophthalmic Services (GOS) 3 supplement the cost of spectacles for children under 16 years. Administrative data on the spectacle refraction dispensed were obtained from Information Services Division (ISD) for mainland Scotland, 2018, and categorised by: Emmetropes/low hyperopes (reference group), myopes and moderate/high hyperopes. Data were linked to the Scottish Index of Multiple Deprivation (SIMD) quintile.

**Results:**

Data included 108, 043 GOS 3 claims. Greater deprivation was associated with greater GOS 3 claims *p* = 0.041. This was most evident in emmetropic/low hyperopic children and in moderate/high hyperopic children. GOS 3 claims in the myopes group increased with age across all SIMD and decreased with age in the moderate/high hyperope group (all *p* < 0.001). GOS 3 claims were not associated with urbanity for all Health Boards (*p* = 0.13).

**Conclusions:**

Children in areas of greater deprivation and in more rural areas are not disadvantaged in accessing NHS spectacles. This did not vary by refractive error group. This suggests that health policy in Scotland is accessible to those from all deprivation levels and refractive errors.

## Introduction

Ocular disease and visual impairment are prevalent amongst populations living in greater deprivation in countries including the UK [1–5]. Furthermore, those living in greater deprived areas are also more likely to present with late-stage eye problems [6–8]. Research in England indicates that health inequality in eye care may be attributed to limited access to optometry services in more deprived areas [9].

Despite the provision of free and universally accessible eye examinations in Scotland, there is some evidence to suggest an underrepresentation of Scottish adults with a lower income or lower education attending an eye examination [1]. It has also been suggested that Scottish health policy has not reached the more vulnerable members of society who are likely to be more affected by greater deprivation, potentially leading to a widening of eye health inequalities in Scotland [1]. There are also fewer children living in more deprived areas attending an NHS eye examination in England [10]. This lack of attendance at eye examinations may translate into a greater proportion of the population in more deprived areas living with uncorrected refractive error. This is a particular concern for children whose eyesight is still at the developmental stage and may be adversely affected by under- or uncorrected refractive error.

Visual screening in children aims to detect visual problems such as reduced vision and amblyopia. Such programmes may assist in reducing ocular inequality, but a recent report by the UK National Screening Committee concludes there is insufficient evidence to support the clinical effectiveness of visual screening in young children aged 4–5 years. However, the report also concludes that the prevalence of amblyopia is lower in populations undergoing vision screening and there is also insufficient evidence to support a change to the methods used within the current vision screening programme [11]. Visual screening programmes may not be sensitive enough to detect visual problems due to their reliance on uncorrected distance visual acuity [12]. Yet, uncorrected refractive error, particularly hyperopia, in children may result in amblyopia, strabismus and a failure to achieve normal vision [13]. It may also negatively impact educational attainment [14] and the development of visuocognitive and visuomotor skills [15]. Hyperopia affects up to a quarter of children in some areas of the UK [16] and is also thought to be greater in children living in more deprived areas [17].

Literature exploring the association between myopia and deprivation level is inconclusive; most studies indicate that there is an association with less deprivation likely due to educational attainment of the parents and higher income [18–21] however other studies report no association with deprivation [22, 23]. This highlights the need for full eye examinations in children from all backgrounds to identify uncorrected refractive errors. Research evidence in this area is scarce and it is unclear if the proportion of children with refractive errors accessing NHS spectacles is linked to the level of deprivation. More research is also needed to evaluate the suggested inefficacy of Scottish eye health policy and whether the argument that current eye care policy increases inequalities can be upheld.

The primary aim of this study was thus to further explore indicators of inequality in Scottish eye care, specifically the proportion of children accessing NHS spectacles in relation to level of deprivation, refractive error, urbanity and age group in mainland Scotland.

## Materials (subjects) and methods

Routinely collected administrative NHS health data were obtained from the Information Services Division (ISD) [24] (now part of Public Health Scotland), consisting of GOS 3 payment claims from all optometric practices in Scotland for children aged 6–15 years. GOS 3 forms are submitted to NHS National Services by Optometric practices to claim a set fee of £39.10 to £215.50, depending on the complexity of the spectacle prescription, to supplement the cost of spectacles for children aged under 16 years in addition to other adult exemption groups. GOS 3 forms are issued if there is a new or changed prescription or if spectacles are unserviceable after a 2-year period. These forms contain data on the spectacle refraction dispensed. This comprehensive dataset provided the number of GOS 3 claims by year of age and by NHS Health Board for every child who accessed NHS funded spectacles in all of Scotland. Although small levels of refractive error may not be detected without an eye examination, these data are likely to represent most children with symptomatic refractive errors.

All GOS 3 data were linked to Scottish Index of Multiple Deprivation (SIMD), a national population-based tool, using the postcode the child lives at. The SIMD combines 38 domains including income, education, housing and crime [25] and is ranked in quintiles from most deprived (rank 1) to least deprived (rank 5). Each rank accounts for 20% of the population in each NHS Health Board by year of age. The data linkage was facilitated by ISD to ensure accuracy and participant anonymity.

To summarise, data included the number of GOS 3 claims submitted in each of the 14 NHS Health Boards [26] for children aged 6–15 years for each SIMD quintile for the year 2018.

Due to the dataset consisting of secondary data, ethical approval was not required for this study.

### Statistical analyses

Spherical equivalent refraction (SER), defined as sphere + (0.5*cyl), was used to categorise participants into refractive groups [16, 27]. Emmetropia/low hyperopia was defined as SER −0.25DS to <+2.00DS, myopia was defined as SER ≤ −0.50DS and moderate hyperopia was defined as SER ≥ +2.00DS.

A single payment claim per patient and year was analysed. Where more than one GOS 3 claim was submitted to NHS National Services for the same patient, data for the most recent claim were used. To provide a comparable estimate of GOS 3 claims for each NHS Health Board area, we calculated the percentage of GOS 3 claims per total population for each NHS Health Board by SIMD, using population data for 2018 for children aged 6–15 years from National Records of Scotland [28].

To ensure confidentiality and adherence to data protection regulations, data from three NHS Health Boards were excluded (NHS Orkney, NHS Shetland, NHS Western Isles). Thus the results pertain to mainland Scotland, providing information on the region in which the large majority of the population resides. A small number of data on mainland Scotland were also not accessible from ISD due to low numbers. This equated to 1.6% of the total data for emmetropes/low hyperopes; 3.8% of the total data for myopes and 2% of the total data for moderate/high hyperopes.

A multiple variable adjusted regression model using robust standard errors and interaction terms was used to explore the association between the proportion of GOS 3 claims in each refractive group, age and SIMD. The emmetrope/low hyperope group and SIMD 1 were included as the reference groups. The coefficient, *p* value and confidence intervals (CIs) are reported. A likelihood ratio test was performed to determine if inclusion of interaction terms (SIMD, age and refractive group) improved the fit of the model.

The percentage of the population living in an urban area for each NHS Health Board was determined using data from National Records of Scotland [29]. An urban area was defined as settlements of 3000 people or more [29]. The association between the percentage of the population living in an urban area and the proportion of GOS 3 claims for each NHS Health Board was explored using linear regression.

Data analyses were performed using Stata 13.1 (StataCorp TX, USA) using a statistical significance level of 5% (*p* < 0.05).

## Results

### Relative distribution of number of GOS 3 claims across SIMD and refractive groups with age

A total of 108, 043 GOS 3 claims were available for analysis from across all 11 NHS Health Boards in mainland Scotland.

**Fig. 1:**
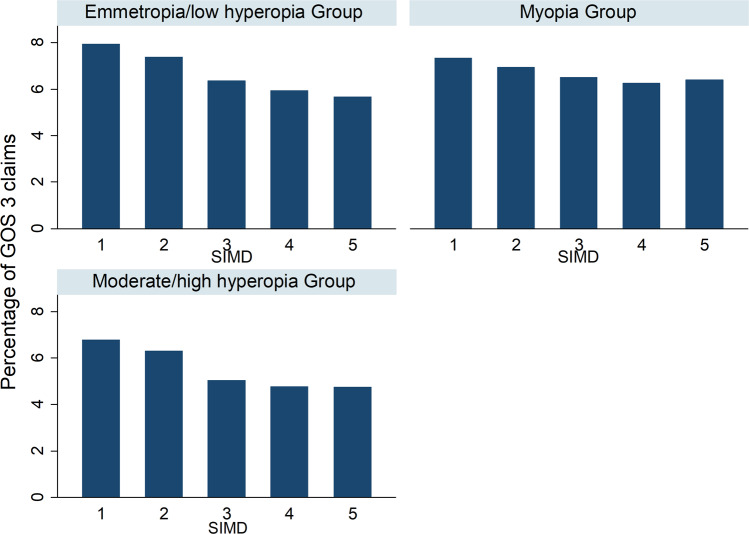
The distribution of the average percentage of GOS 3 claims relative to total population in each SIMD by refractive group in children aged 6–15 years.

For the cohort as a whole, there was an association between increasing GOS 3 claims and decreasing SIMD (Table 1). Figure 1 illustrates the association between GOS 3 claims and SIMD for each refractive group. Across the three refractive groups, a very similar pattern was observed in that marginally more GOS 3 claims were made in most deprived (quintile1, left) as compared to less deprived areas (quintile 5, right). There was a significant interaction effect between SIMD and the myopia group with GOS 3 claims increasing between SIMD 4 and 5. There was a significant interaction between SIMD and refractive groups (*p* = 0.041) (Table 1).

**Table 1:** Multiple variable adjusted regression results pertaining to the association between the percentage of GOS 3 claims out of the total population as the independent variable and SIMD, age and refractive category.

Variable	Coefficient	*p*	96% Confidence interval
SIMD			
1			
2	−0.56	0.02	−1.03 to −0.09
3	−1.56	<0.001	−1.99 to −1.13
4	−1.98	<0.001	−2.39 to −1.58
5	−2.27	<0.001	−2.69 to −1.86
Refractive group			
Emmetropes/low hyperopes			
Myopes	−12.76	<0.001	13.57 to −11.95
Moderate/high hyperopes	2.60	<0.001	1.74 to 3.47
Interaction between SIMD and refractive group			
Emmetropes/low hyperopes			
SIMD 2 and myopes	0.31	0.33	−0.32 to 0.95
SIMD 2 and moderate/high hyperopes	0.10	0.76	−0.55 to 0.76
SIMD 3 and myopes	0.90	0.004	0.29 to 1.51
SIMD 3 and moderate/high hyperopes	−0.15	0.63	−0.77 to 0.46
SIMD 4 and myopes	1.04	<0.001	0.46 to 1.63
SIMD 4 and moderate/high hyperopes	−0.01	0.96	−0.60 to 0.57
SIMD 5 and myopes	1.42	<0.001	0.85 to 1.99
SIMD 5 and moderate/high hyperopes	0.23	0.44	−0.36 to 0.83
Likelihood ratio test (SIMD × refractive group)	*p* = 0.041		
Interaction between age and refractive group			
Emmetropes/low high hyperopes			
Age and myopes	1.13	<0.001	1.07 to 1.20
Age and moderate/high hyperopes	−0.36	<0.001	−0.42 to −0.29
Likelihood ratio test (age × refractive group)	*p* < 0.001		
Likelihood ratio test (age × refractive group × SIMD)	*p* < 0.001		

*SIMD* Scottish Index of Multiple Deprivation, *CI* Confidence Interval.

**Fig. 2:**
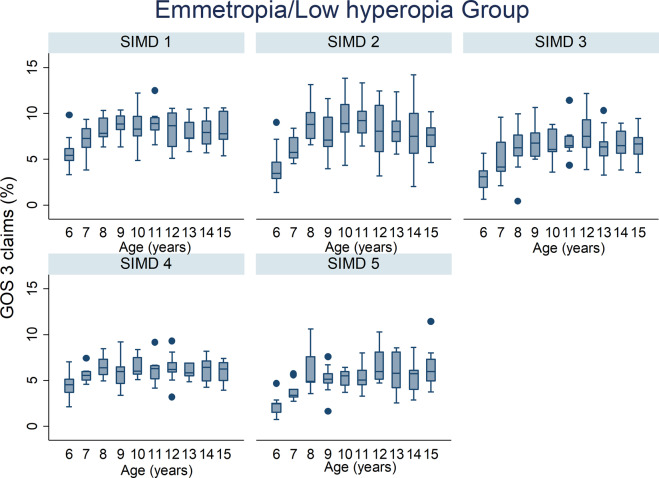
Box plots illustrating the percentage of GOS 3 claims out of the total population and age in the emmetrope/low hyperope group for all NHS health boards. The box plots display the median value and the 25th and 75th percentiles for each year for age for each SIMD.

**Fig. 3:**
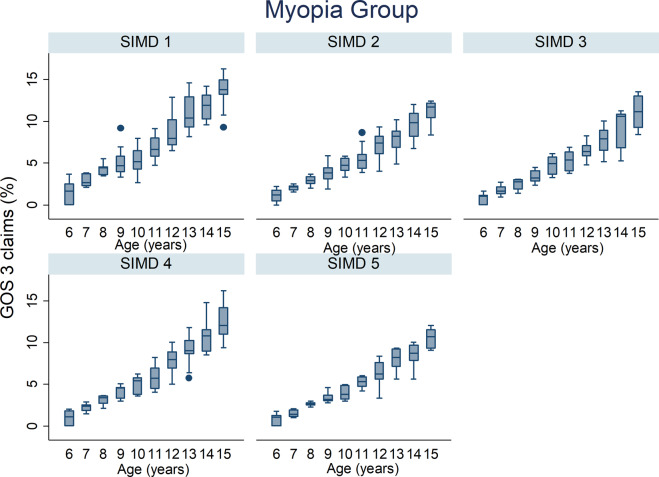
Box plots illustrating the percentage of GOS 3 claims out of the total population and age in the myopia group for all NHS health boards. The box plots display the median value and the 25th and 75th percentiles for each year for age for each SIMD.

**Fig. 4:**
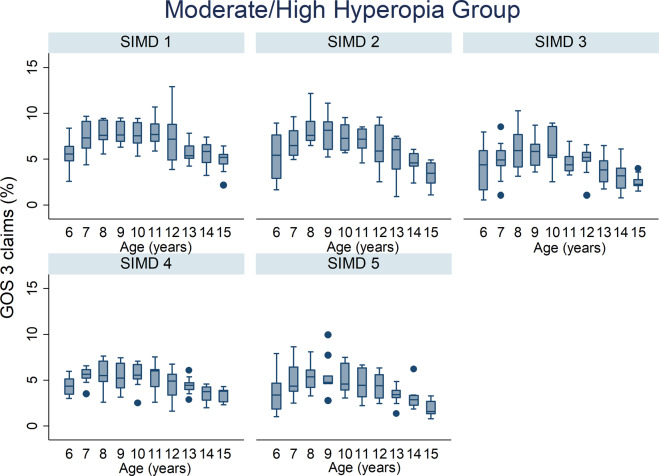
Box plots illustrating the percentage of GOS 3 claims out of the total population and age in the moderate/high hyperopic group for all NHS health boards. The box plots display the median value and the 25th and 75th percentiles for each year for age for each SIMD.

The percentage of GOS 3 claims by age across all deprivation categories for all refractive group is illustrated in Figs. 2–4. The percentage of GOS 3 claims increased significantly with age in myopic children (*p* < 0.001, Table 1). The opposite finding was observed for moderate/high hyperopic children, where fewer claims were made for older children (*p* < 0.001, Table 1). There was a significant interaction effect between refractive group and age which improved the fit of the statistical model (all *p* < 0.001).

### The association between urbanity and proportion of GOS 3 claims

The percentage of the population residing in an urban area ranged from 38.3% (NHS Tayside) to 98.0% (NHS GGC). There was no association between the proportion of GOS 3 claims and percentage of the population living in an urban area for all of the NHS Health Boards (Coef: 0.09, *p* = 0.13, 95% CI: −0.03 to 0.20).

## Discussion

This is the first study in the UK to explore the proportion of children accessing NHS spectacles from different deprivation backgrounds and with differing refractive errors. This large dataset provides three novel key findings. The first is that children with any type of refractive error and across all socioeconomic groups are accessing primary eye care and are provided with spectacles in Scotland. Second, there are age-related differences in the proportion of children receiving spectacles for myopia, with more GOS 3 claims being submitted for older children with myopia. Third, there was no association between the percentage of GOS 3 claims and urbanity of the NHS Health Board. This can be interpreted as an indication that primary eye care in areas with considerable rural geography is as accessible as eye care in urban regions.

Optometry services in the UK are primarily business entities in community settings. Although practices receive fees for eye examinations from the National Health Service (NHS), they rely on spectacle and contact lens sales to ensure viability [30]. It is therefore unsurprising that some optometry practices may be more frequently located in areas of greater wealth and less deprivation as reported in England [9]. However, a recent report in Scotland found no difference in the distribution of optometry practices sampled from across nine NHS Health Boards [31]. This even distribution of optometry practices across deprivation areas may contribute to the current finding that children from all socioeconomic backgrounds are accessing NHS spectacles.

Results indicated that there was a slightly greater proportion of children, particularly those with more hyperopic refractive errors and with emmetropic/low hyperopic, refractive errors accessing NHS spectacles in more deprived areas. This finding may indicate that there are more children with hyperopic refractive errors in more deprived areas in agreement with research in the UK [17]. The percentage of children with more hyperopic refractive errors aged 6–7 years (5.5%) and aged 12–13 years (5.3%) is lower than the reported prevalence of hyperopia determined by cycloplegic autorefraction in Northern Ireland in children of the same ages (21.7% and 14.2%, respectively) [32]. However, the current data for children aged 12–13 years are similar to the prevalence of hyperopia, by cycloplegic autorefraction, in children of the same age in England (5.4%) [27].

It is unknown if cycloplegic refraction was completed for every child from the current administrative dataset and therefore the proportion of younger hyperopic children requiring spectacles may be underrepresented. However, with greater public awareness, free NHS eye examinations, and the reporting of visual symptoms by teachers, parents and even young children [33], it likely that only a small proportion of hyperopic children will not have been represented in the current study. Furthermore, cycloplegic refraction during a routine eye examination in children is commonplace in community optometry practices in Scotland.

The percentage of children with myopic refractive errors aged 6-7 years in the current study (1.7%) is similar to the reported prevalence of myopia in children of the same age in Northern Ireland (1.9%) [32]. In children aged 12–13 years, the percentage of children with myopic refractive errors in the current study is 8.9% is slightly less than the reported prevalence of myopia in this age group in Northern Ireland (14.6%) [32]. By the age of 15 years, the percentage of children accessing spectacles with myopic refractive errors increased to 13.2% in the current study which is the same as the reported prevalence of myopia in Norwegian adolescents [34]. Therefore, it is likely that the proportion of myopic children determined from this administrative dataset to be similar to the expected prevalence of myopia in Scotland. This is further supported by research in the UK which reports that the use of unaided visions in pre-school screening is sensitive at detecting myopia in children [12].

The provision of GOS 3 services in Scotland is successful in ensuring that children from all socioeconomic backgrounds, from both rural and urban backgrounds and with a variety of refractive errors can access NHS spectacles. This indicates that health policy in Scotland may aid in reducing ocular health inequalities. The increase in the number of children with myopia with age and the proportion of adolescents with myopia in the current study is similar to data reported in comparable populations.

## Summary

### What was known before

- Adults living in deprived areas have more advanced eye disease and may be less likely to attend an eye examination.

### What this study adds

- Children with differing refractive errors living in deprived areas are not disadvantaged in accessing NHS spectacles in Scotland.
- The proportion of children receiving NHS spectacles for myopia increases with age.
- Children living in rural areas are not disadvantaged in accessing NHS spectacles in comparison to children living in urban areas.
